# Increased Frequency of Memory CD4+ T-Cell Responses in Individuals With Previously Treated Extrapulmonary Tuberculosis

**DOI:** 10.3389/fimmu.2020.605338

**Published:** 2020-12-17

**Authors:** Beatriz Barreto-Duarte, Timothy R. Sterling, Christina T. Fiske, Alexandre Almeida, Cynthia H. Nochowicz, Rita M. Smith, Louise Barnett, Christian Warren, Amondrea Blackman, Jose Roberto Lapa e Silva, Bruno B. Andrade, Spyros A. Kalams

**Affiliations:** ^1^Instituto Gonçalo Moniz, Fundação Oswaldo Cruz, Salvador, Brazil; ^2^Multinational Organization Network Sponsoring Translational and Epidemiological Research (MONSTER) Initiative, Salvador, Brazil; ^3^Universidade Salvador (UNIFACS), Laureate Universities, Salvador, Brazil; ^4^Faculdade de Medicina, Universidade Federal do Rio de Janeiro, Rio de Janeiro, Brazil; ^5^Division of Infectious Diseases, Department of Medicine, Vanderbilt University Medical Center, Nashville, TN, United States; ^6^Vanderbilt Tuberculosis Center, Vanderbilt University Medical Center, Nashville, TN, United States; ^7^Faculdade de Medicina, Universidade Federal da Bahia, Salvador, Brazil; ^8^Curso de Medicina, Centro Universitário Faculdades de Tecnologia e Ciências (UniFTC), Salvador, Brazil; ^9^Curso de Medicina, Escola Bahiana de Medicina e Saúde Pública, Salvador, Brazil

**Keywords:** tuberculosis, extrapulmonary tuberculosis, memory T-cell responses, cytokine, CD4+ T cell

## Abstract

Extrapulmonary TB (EPTB) occurs with increased frequency in persons with underlying immunodeficiency. Even after recovery from acute illness, differences in immune phenotype and activation persist. Studies defining characteristics of immune responses after recovery from extrapulmonary TB may provide insights into factors that increase TB risk. We performed two case-control studies (in the United States and Brazil) among HIV-seronegative adults with previous EPTB (n = 9; 25), previous pulmonary TB (n = 7; 25), latent *M. tuberculosis* (Mtb) infection (n = 11; 25), and uninfected TB contacts (n = 10; 25). We assessed the frequency of dual CD4+ interferon-γ and tumor necrosis factor-α responses after stimulation with overlapping Mtb peptides from ESAT-6 or CFP-10, or gamma-irradiated Mtb H37Rv, proliferative responses to Mtb antigens, T-regulatory cell (Treg) frequency and phenotype. In both study populations, individuals with prior EPTB had the highest frequency of intracellular cytokine-producing cells in response to Mtb antigens (p < 0.05; p <.0001). Persons with prior EPTB in Brazil had the highest levels of CD4 proliferation to Mtb antigens (p < 0.0001), and the highest expression of CD39 on Tregs (p < 0.0001). Individuals with treated EPTB maintained high frequencies of Mtb-specific memory responses and active Treg cells, suggesting that susceptibility to EPTB occurs despite the ability to develop and maintain enhanced adaptive immune responses.

## Introduction

The global burden of tuberculosis (TB) is enormous, with an estimated one-quarter of the world’s population (approximately 2 billion people) infected with *M. tuberculosis* (Mtb) and 10 million new cases of TB each year (1). According to the World Health Organization, Mtb is among the leading causes of death due to an infection, causing approximately 1.4 million deaths in 2019 (2).

The immune response to Mtb involves monocytes, macrophages and T-lymphocytes that produce cytokines such as IFN-γ, TNF-α, IL-12, and CXCL8 (IL-8) (3, 4). Over 80% of tuberculosis (TB) disease in low TB incidence settings is due to reactivation of latent Mtb infection (5) yet only 5–10% of persons with latent TB infection (LTBI) progress to TB disease (6–8). The transition from LTBI to active disease could be due to a breakdown in host immune surveillance, a change in the mycobacteria from a dormant to an active state, or a combination of both. There is likely a spectrum of disease activity that includes incipient and sub-clinical disease, in addition to latent/quiescent and active (symptomatic) TB (9). Discovery of factors that predispose to active disease will help identify individuals with LTBI at increased risk for progression to TB so that effective preventive measures can be initiated. Conversely, such information may also help identify protective immune responses that could be induced by TB vaccines.

Extrapulmonary TB (EPTB) is commonly associated with underlying immune defects. Persons with HIV (PWH) are at increased risk of EPTB and this risk increases as the CD4+ T-lymphocyte count declines (10). Young children also have an increased incidence of EPTB, specifically TB meningitis, presumably due to immature immune responses (11). We have previously noted reduced peripheral blood mononuclear cell (PBMC) cytokine production and CD4+ T-lymphocytes in HIV-seronegative adults with previous EPTB compared to persons with previous pulmonary TB or LTBI (12).We also found that persons with previous EPTB had increased T-regulatory (Treg) cell frequency and CD4+ lymphocyte activation, indicating possible immune dysregulation (13).

Active TB disease is associated with increased generalized immune activation, and infiltration of activated T-cells and Tregs at the site of disease (14–19). A sub-population of Tregs (defined as CD4+CD25highFOXP3+) expresses CD39, a surface ectonucleotidase which metabolizes pro-inflammatory extracellular ATP (20). These “active Tregs” exhibit robust and stable suppression of immune function (21). Individuals with active TB have higher frequencies of Treg-expressing CD39 after *in vitro* stimulation with Mtb antigens, and depletion of these cells enhances cytokine secretion in response to Mtb antigens (22, 23). These observations have led to a model whereby secretion of immunomodulatory cytokines such as IL-10 by Tregs impairs Mtb-specific CD4+ and CD8+ T-cell activation and proliferation (24). Studies of Treg frequency during Mtb infection have been limited to individuals with active disease. While it is plausible that expansion of these cells may limit immune responses, and potentially play a role in TB pathogenesis, it is unknown whether high frequencies of Tregs at the time of initial Mtb infection predisposes individuals to develop active TB disease.

The goal of this study was to determine whether features of immune responses could differentiate individuals with LTBI from those with prior treated TB disease. We compared the frequencies of cytokine-producing T-cells and proliferation responses of T-cells stimulated with Mtb peptides (ESAT-6 and CFP10) and gamma-irradiated Mtb in persons with previous extrapulmonary or pulmonary TB disease, LTBI and uninfected individuals who had been exposed to TB. We also measured surface expression of CD39 on Tregs as a possible marker of regulatory function. Two case-control studies were performed—one in the United States and one in Brazil. The correlates of protection from active disease are unknown, but analysis of immune parameters among persons who have recovered from acute illness could provide clues into factors that increase TB risk.

## Methods

### Design of the Case-Control Studies

For both case-control studies, cases were defined as persons with previously treated extrapulmonary TB. There were three sets of controls: 1) persons with previously treated pulmonary TB, 2) persons with LTBI (defined as a tuberculin skin test (TST) >5 mm induration or positive interferon gamma release assay (IGRA); regardless of whether they had previously been treated), and 3) persons who had been exposed to culture-positive pulmonary TB but were not infected (i.e., TST <5 mm induration or negative IGRA). Persons with both pulmonary and extrapulmonary TB were considered extrapulmonary for the purposes of analysis, given the presence of disseminated disease. Inclusion criteria consisted of: age ≥18 years at time of diagnosis of TB disease or infection; HIV-seronegative; culture-confirmed disease and either near completion (within one month) or after completion of therapy (for extrapulmonary TB cases and pulmonary TB controls). We did not require persons to complete therapy for LTBI to be enrolled because such persons are asymptomatic and latent Mtb infection is unlikely to alter systemic cytokine production, unlike active TB disease. Only contacts of culture-positive pulmonary TB cases were included as controls—both those with and without evidence of Mtb infection. Contacts of culture-positive pulmonary TB cases were tested for LTBI at the beginning of the contact investigation and 8–12 weeks later if the initial test was negative (25). Exclusion criteria consisted of: serum creatinine >2 mg/dL; use of corticosteroids or other immunosuppressive agents at the time of diagnosis or study entry; malignancy; and diabetes mellitus. HIV-positive persons were excluded because of the known increased risk of extrapulmonary TB associated with HIV/AIDS (10, 26, 27). Pleural TB has exaggerated (not diminished) local cell-mediated immune responses (28) Although it is unclear whether this affects the systemic immune response, we excluded persons with previous pleural TB because they may exhibit a unique immunopathogenesis compared with the other forms of extrapulmonary (disseminated) TB.

In the U.S. study population, all cases and controls were enrolled from Tennessee. Extrapulmonary TB cases and pulmonary TB controls were identified by review of the Tennessee Department of Health TB registry. Ongoing contact investigations at local and regional TB clinics were reviewed to identify patients in the remaining control groups. Demographic and clinical characteristics were collected from the patient or the Tennessee TB registry.

### United States Study: Sample Preparation

Peripheral blood mononuclear cells (PBMC) were isolated under sterile conditions by Ficoll-Paque (GE Healthcare Bio-Science) density gradient centrifugation and cryopreserved in 90% of fetal bovine serum (FBS) (GemCell-Gemini Bioproducts) and 10% DMSO (Dimethyl sulfoxide) (29). Blood samples were submitted to a commercial laboratory for HIV serology and complete blood counts.

### In Vitro Stimulation of PBMC for Intracellular Cytokine Staining

Peripheral blood mononuclear cells (PBMCs) from each subject were thawed, and cultured at 10 x10^6^ cells/ml in 48-well plates (2 x 10^6^ cells/well) in R10 medium (RPMI 1640 containing 10% heat inactivated FCS, 2 mM L-glutamine, 50 µg/ml penicillin, 50 µg/ml streptomycin, and 10mM Hepes) and co-stimulated with anti-CD28 (1µg/ml, BD Biosciences) and anti-CD49d (1µg/ml, BD). Cells were stimulated with overlapping M. tuberculosis peptides from the ESAT-6 or CFP-10 proteins (BEI Resources) (2 µg/ml), or gamma-irradiated h37RV (10 µg/ml) (BEI Resources). As a negative control, PBMCs were incubated with media alone and as a positive control with Staphylococcal Enterotoxin B (SEB) (1 µg/ml, Sigma). One hour after stimulation, brefeldin was added; after overnight incubation, PBMCs were recovered, washed, and stained with the appropriate antibodies. Cytometry was performed (BD LSRFortessa) at the Vanderbilt University Medical Center Flow Cytometry Shared Resource and analyzed using FlowJo v10.0.8 (Tree Star).

### Flow Cytometry Antibody Panels

Antibodies for intracellular cytokine staining included: anti-CD3-AF700, CD4-PcPCy5.5, CD8-PECF594, IFNg-FITC, CD14-V500, CD19-V500, TNFα-APC, CD38-PECy7, HLADR-BV605 (BD Biosciences); PD-1-PE, IL-2-BV421 (BioLegend); Live-Dead-AquaViD (LifeTechnologies).

The T regulatory cell/activation panel included: anti-CD3-AF700, CD25-PE, HLA-DR-FITC (BD); CD4-PETxR, CD8-APC AF750, Live-Dead-AquaViD (LifeTechnologies); CD39-PECy7, FOXP3-APC (eBioscience); CD127- PE Cy5.5 (Beckman Coulter); CD38- BV421 (BioLegend).

### Cell Proliferation Assay

PBMC were thawed, washed with PBS, and labeled with CellTrace Violet (Life Technologies) at a final concentration of 5 µM. Cells (2 x 10^6^ cells per condition) were incubated with Media, SEB 1 µg/ml, gamma-irradiated h37rv (10 µg/ml), ESAT-6 peptide pool (2 µg/ml per peptide), CFP-10 peptide pool (2 µg/ml per peptide). IL-2 at a final concentration of 1 U/ml was added at day 3. On day 6 cells were washed, and stained with anti –CD3, -CD4, -CD8, -CD14/CD19 (dump channel), and the % of CellTrace Violet low cells was measured.

### The Brazilian Case-Control Study

In the Brazilian study population, cryopreserved PBMC samples and corresponding clinical and epidemiological data were obtained from participants enrolled in a translational study performed at the Instituto Brasileiro para Investigação da Tuberculose (IBIT) and at the Hospital Especializado Octavio Mangabeira (HEOM), Salvador, Bahia, northeast Brazil, between December 2015 and January 2018 (30). All persons with extrapulmonary TB had lymphatic disease.

The parent study was focused on characterization of inflammatory markers in different clinical forms of TB and recruited 1,792 individuals with presumptive TB at the referral primary care clinics. Participants underwent clinical assessments and chest x-ray examination. In addition, acid-fast bacilli (AFB) screening in sputum smears (by microscopy) and sputum cultures (Lowenstein–Jensen solid cultures) was performed in all patients. The patients with active TB were treated following the Brazilian National Guidelines (31). The parent study collected 10 ml of venous blood in sodium heparin tubes for isolation of PBMCs from a subset of participants who consented to blood collection. Blood collection occurred prior to initiation of anti-TB therapy (baseline), at month 2 and month 6 of treatment, and at month 18 after enrollment. Cells were cryopreserved in liquid nitrogen at the biorepository of the Laboratory of Inflammation and Biomarkers, Fundação Oswaldo Cruz, Salvador, Brazil.

The parent study also included participants who were asymptomatic contacts of pulmonary TB index cases. All TB contacts were actively screened for TB through clinical, radiologic, and microbiologic investigation (32). At the time of study enrollment, individuals not living with HIV who tested positive for QuantiFERON TB Gold-in-Tube (QFT) enzyme-linked immunosorbent assay (Qiagen) and also exhibited a positive tuberculin skin test (TST) result (> 5 mm) were considered to have latent TB infection (LTBI), and individuals who were QFT-negative and had negative TST result (< 5 mm) were considered uninfected controls (30).

The present investigation was a sub-study focused on characterization of T-cell responses at month 18 after enrollment (approximately 1 year after patients achieved microbiologic cure) in patients with drug-sensitive PTB or EPTB. We selected individuals with confirmed pulmonary or extrapulmonary TB, matching by age (± 5 years) and sex, as well as for controls with or without LTBI. Samples from TB patients >18 years old, HIV-negative, and no treatment failure, abandonment or relapse were included in the study. The exclusion criteria were the same used for the sub-study performed in USA.

### Flow Cytometry in the Brazilian Study

Cryopreserved PBMCs were thawed and resuspended in 1640 Roswell Park Memorial Institute medium supplemented with 10% fetal bovine serum at 10^6^ cells per well in 96-well plates and rested for 2 h at 37°C in 5% CO2. Cells were washed and resuspended in complete media with Brefeldin-A (Biolegend, San Diego, CA) and Monensin (Biolegend, San Diego, CA) to block cytokine secretion and stimulated with ESAT-6 and CFP-10 peptide pools (10 μg/ml) or irradiated H37Rv strain of M. tuberculosis (MOI: 5) (all from BEI Resources) overnight at 37°C in 5% CO2. Cells were then stained for cell surface markers with the following panel of antibodies: CD3 (clone OKT3), CD4 (clone RPA-T4), CD8 (clone OKT8), CD127 (clone eBioRDR5), CD25 (clone PC61.5), CD39 (clone eBioA1), and PD1 (clone J43), all from ThermoFisher. Cells were then fixed and permeabilized using the Foxp3 Fixation and Permeabilization Buffer (eBioscience). Intracellular staining was performed to detect IFN-γ (clone 4S.B3), TNF-α (clone Mab11), and all from ThermoFisher. In parallel experiments, we performed cell proliferation assay using the CellTrace Violet Cell Proliferation Kit (ThermoFisher) following the manufacturer’s protocol. Acquisition of stained cells was performed using a BD LSRFortessa cell analyzer (BD Bioscience, San Jose, CA) and analyzed using FlowJo software (BD Bioscience, San Jose, CA).

### Statistical Analysis

In the U.S. study population, a convenience sample of available specimens from a previous study was used (33). In the Brazil study population, sample size was determined based on power of 80% (alpha error, 5%) to detect differences in median frequencies of T-cell subsets >2.5% between EPTB cases and TST negative controls, based on a previous study from our group (30).

Categorical variables were compared using the Pearson’s chi-square test. Distributions of continuous variables were compared using the rank-sum and Kruskal-Wallis tests with Dunn’s multiple comparisons *ad hoc* test. The chi-square test for trend was used to evaluate the fraction of responders across groups. Two-sided p values of <0.05 were considered statistically significant. Statistical analyses were performed using Graphpad Prism 8.0 (GraphPad Software, San Diego, CA).

## Results

In the U.S. study population, there were 10 controls without LTBI, 11 controls with LTBI, 7 pulmonary TB controls, and 9 extrapulmonary TB cases. In the Brazilian study population, there were 25 participants in each of the four patient groups. The clinical and demographic characteristics of the study populations are in **Tables 1** and **2**. In both study populations, cases and controls were similar according to age, sex, tobacco use, and alcohol use. In the U.S. study population, participants with LTBI were more likely to be of black race, and persons with extrapulmonary TB were more likely to be born outside of the United States. Both extrapulmonary TB cases and pulmonary TB controls were evaluated a median of at least one year after TB treatment completion.

**Table 1:** Clinical and demographic characteristics of the study population in the United States (Tennessee).

Characteristic	No LTBI	LTBI	Prior PTB	Prior EPTB	P-value
N	10	11	7	9	
Age (years), median (IQR)	49 (30,52)	51 (39,57)	50 (38,66)	39 (36,45)	0.66
Male sex, no. (%)	6 (60)	6 (55)	6 (86)	5 (56)	0.55
Hispanic ethnicity, no. (%)	0 (0)	1 (9)	1 (14)	2 (22)	0.49
Black race, no. (%)	3 (30)	7 (64)	0 (0)	2 (22)	0.02
Foreign born, no. (%)	1 (10)	2 (18)	1 (14)	6 (67)	0.03
Tobacco use^1^, no. (%)	2 (20)	3 (27)	5 (71)	2 (22)	0.13
Alcohol use^2^, no. (%)	4 (40)	2 (18)	2 (29)	1 (11)	0.54
Years from treatment completion to blood draw (years), median (IQR)	N/A	N/A	1.2 (0.60,2.80)	1.7 (0.94, 4.0)	0.34

LTBI, latent tuberculosis infection, based on a positive tuberculin skin test; PTB, pulmonary tuberculosis;

EPTB, extrapulmonary tuberculosis.

Data are shown as median and interquartile (IQR) range or frequency (percentage). Data were compared between the clinical groups.

Using the Kruskal-Wallis test (continuous variables) or the Pearson’s χ 2 test (for data on frequency).

^1^More than 10 cigarettes/day;

^2^Four or more drinks/week;

N/A Not applicable.

**Table 2:** Clinical and demographic characteristics of the study population in Brazil (Bahia).

Characteristic	No LTBI	LTBI	Prior PTB	Prior EPTB	P-value
N	25	25	25	25	
Age (years), median (IQR)	24 (20–31)	26 (20–33)	28 (19–31)	26 (21–28)	> 0.99
Male sex, no. (%)	13 (52)	12 (48)	13 (52)	14 (56)	0.96
Non-white race, no. (%)	22 (88)	20 (80)	24 (96)	23 (92)	0.31
Illicit drug use^1^ no. (%)	2 (8)	3 (12)	4 (16)	3 (12)	0.86
Smoking^2^ no. (%)	1 (4)	3 (12)	7 (28)	3 (12)	0.10
Alcohol abuse^3^ no. (%)	5 (20)	7 (28)	10 (40)	14 (56)	0.08
Acid-fast bacilli smear grade no. (%)					0.58
0	25 (100)	25 (100)	0 (0)	0 (0)	
1+/scanty	0 (0)	0 (0)	1 (4)	3 (12)	
2+	0 (0)	0 (0)	14 (56)	13 (52)	
≥3+	0 (0)	0 (0)	10 (40)	9 (36)	

LTBI, latent tuberculosis infection, based on a positive tuberculin skin test; PTB, pulmonary tuberculosis; EPTB, extrapulmonary tuberculosis.

Data are shown as median and interquartile (IQR) range or frequency (percentage). Data were compared between the clinical groups using the Kruskal-Wallis test (continuous variables) or the Pearson’s χ 2 test (for data on frequency).

Mycobacterium tuberculosis from either sputum or extrapulmonary site; all extrapulmonary disease was lymphatic.

The frequency of individuals with different values of acid-fast bacilli smear grade at the time of diagnosis was compared between PTB and EPTB groups (the groups of individuals without and with LTBI, as well as persons with negative smears, were excluded from this analysis).

Smear grade was from sputum samples for PTB patients or lymph node aspirates for EPTB.

All individuals tested negative for HIV infection.

^1^Illicit drugs: cannabis, cocaine, or crack;

^2^Past or current cigarette smoking;

^3^Two or more points on the CAGE questionnaire.

### Individuals With Prior Extrapulmonary TB Had the Highest Level of Intracellular Cytokine Responses to *M. tuberculosis* Antigens

**Figure 1:**
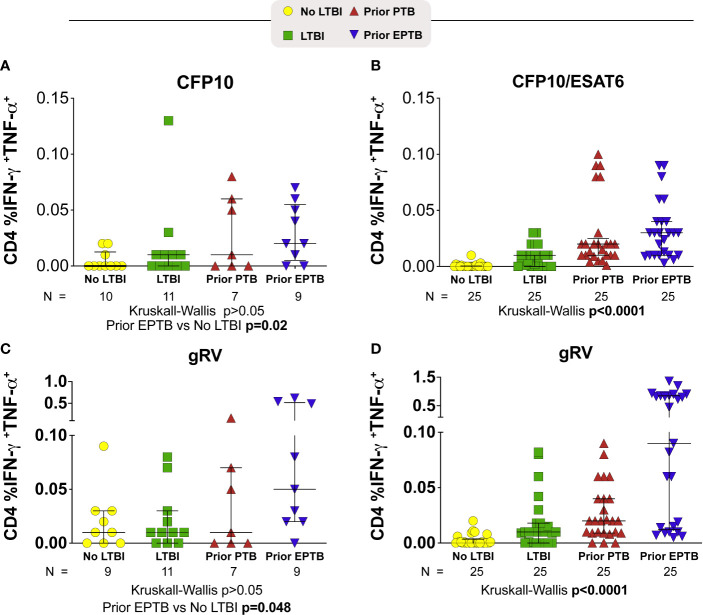
CD4+ T-cell intracellular cytokine responses to M. tuberculosis antigens. Scatter plots depicting the expression of intracellular cytokine by CD4+ T-lymphocytes from TB patients with PTB or EPTP presentation and controls (TST+ and TST-). Frequencies of IFN-γ+TNF-α+CD4+ T cells from whole blood obtained and compared according to the stimulation. **(A, B)** Frequencies of IFN-γ+TNF-α+CD4+ T cells responses to stimulation with a pool of CFP-10 peptides (A; U.S. study population) or CFP-10 and ESAT-6 (B; Brazilian study population). **(C, D)** Frequencies of IFN-γ+TNF-α+CD4+ T cells responses after incubation of PBMC with gRV (C; U.S. study population) (D; Brazilian study population). Background has been subtracted out. Lines represent median values and interquartile ranges (IQR). The differences in median values (and IQR) between groups were compared using the Kruskal-Wallis test with Dunn’s multiple comparisons post-test. TB, tuberculosis; PTB, pulmonary tuberculosis; EPTB, extrapulmonary tuberculosis; TST, tuberculin skin test.

We evaluated cytokine responses to overlapping peptides from the ESAT-6 and CFP-10 proteins as well as whole lysate of inactivated (gamma-irradiated) Mtb (gRV) (**Figure 1**). In the U.S. study population in response to the CFP10 peptide pool, the median frequency of interferon (IFN)-γ and TNF-α producing cells tended to be highest among those with prior EPTB (P = >0.05; Kruskal-Wallis) (**Figure 1A**). Compared to controls without LTBI, persons with prior EPTB had the highest magnitude response (P = 0.02; Dunn’s posttest). In the Brazilian study population in response to CFP10 and ESAT6, persons with prior EPTB had the highest IFN-γ and TNF-α producing cells (P < 0.001; Kruskal-Wallis) (**Figure 1B**). Similar trends of higher responses in persons with prior EPTB was observed in response to inactivated Mtb organisms, in both study populations (**Figures 1C, D**).

### Individuals With Prior Extrapulmonary TB Had High Levels of CD4+ Proliferation in Response to *M. tuberculosis* Antigens

**Figure 2:**
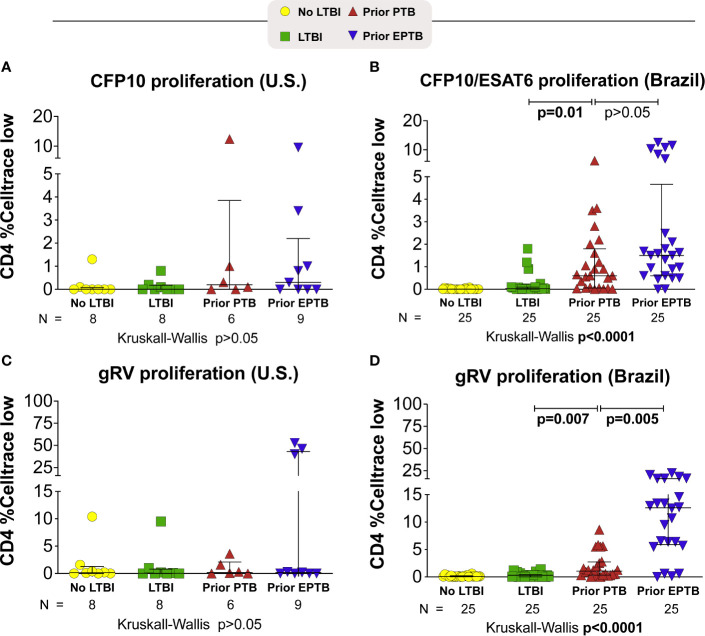
Proliferative responses to M. tuberculosis antigens. Briefly, PBMC were incubated with *M. tuberculosis* antigens for 6 days. Proliferating cells were identified as Celltrace violet low. **(A, B)** Celltrace low expression in CD4+ T-cells stimulated with CFP-10 pool or CFP-10 pool and ESAT-6 **(A)** U.S. study population. **(B)** Brazilian study population. **(C, D)** Celltrace low expression in CD4+ T-cells stimulated with gRV. Lines represent median values and interquartile ranges (IQR). The differences in median values (and IQR) between groups were compared using the Kruskal-Wallis test with Dunn’s multiple comparisons post-test. TB, tuberculosis; PTB, pulmonary tuberculosis; EPTB, extrapulmonary tuberculosis; TST, tuberculin skin test; CFP, Culture filtrate protein; ESAT, early secretory antigen of tuberculosis; gRV, γ-irradiated *M. tuberculosis*.

In Brazil, persons with prior EPTB had the highest levels of CD4 proliferation to M. tuberculosis antigens (P < 0.0001) (**Figure 2**). Although not seen to the same extent in the U.S. population, among persons with prior EPTB there were 3 outliers with high levels of CD4 proliferation.

### T Regulatory Cell Frequency Phenotype Among Individuals

**Figure 3:**
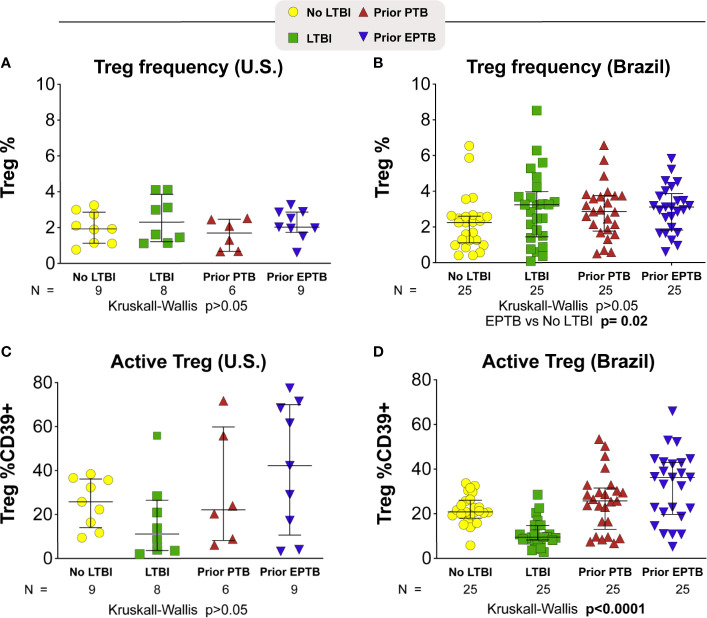
T-regulatory cell frequency and phenotype. Scatter plots depicting the Treg frequency **(A)** in U.S. study population **(B)** in Brazilian study population. Frequency of Treg CD39+ **(C)** in U.S. study population **(D)** in Brazilian study population. Lines represent median values and interquartile ranges (IQR). The differences in median values (and IQR) between groups were compared using the Kruskal-Wallis test with Dunn’s multiple comparisons post-test. TB, tuberculosis; PTB, pulmonary tuberculosis; EPTB, extrapulmonary tuberculosis; TST, tuberculin skin test; CFP, Culture filtrate protein; ESAT, early secretory antigen of tuberculosis; gRV, γ-irradiated *M. tuberculosis*.

We measured both Treg cell frequency and expression of the CD39 marker on Treg and non-Treg CD4+ T cells in these individuals (**Figure 3**). In the U.S. population we found no significant differences in Treg frequencies across groups (p > 0.05; Kruskal-Wallis; **Figure 3A**). While the individuals with the highest expression of CD39 were in the EPTB group, the median expression was not statistically significant across groups (**Figure 3C**). We also measured PD-1 expression and saw no trend across groups (data not shown).

In the Brazil study population, Treg CD39 expression was the lowest in the LTBI group, and highest in the EPTB group overall (p < 0.0001; Kruskal-Wallis; **Figure 3D**). Indeed, Treg CD39 expression among persons with LTBI was lower than that in the no LTBI (p = 0.0003); PTB (p = 0.0001) and EPTB groups (p = 0.0001) after adjustment for multiple comparisons. Although PTB had lower than EPTB Treg CD39 expression, this was not statistically significant (p > 0.05).

## Discussion

We sought to determine whether functional differences in immune responses were present among individuals after successful treatment of pulmonary or extrapulmonary TB. Most studies describing immune function in the setting of differing manifestations of TB have compared individuals with active disease to individuals with LTBI (34, 35). Chronic infection is associated with changes in immune function due to chronic activation and subsequent immune exhaustion (23). In a prior study, we found that even several months to years after clearance of TB disease, individuals with prior extrapulmonary TB had persistently elevated immune activation and increased frequencies of circulating Treg cells (13). Here, we extended this analysis to evaluate the frequencies of Mtb-reactive T cells and perform a more detailed phenotypic analysis of circulating Treg cells.

We found levels of Mtb-reactive responses to be highest in persons with previous EPTB. This finding was consistent in both the U.S. and Brazilian study populations. These cells were polyfunctional, as they were able to secrete IFN-γ and TNF-α. Our findings contrast with Scriba et. al, who found lower frequencies of Mtb-reactive cells in individuals with self-reported previously treated TB disease (36). This may be because we included a group with confirmed prior extrapulmonary TB, while in the other study the location of TB disease was not specified. Future studies to evaluate the immune factors associated with control of M. tuberculosis infection should specifically study the extent of active disease.

In our prior work, we found increased levels of immune activation and Treg frequency in individuals with prior extrapulmonary TB. In this smaller North American cohort, we found a similar trend toward higher frequencies of Treg cells in individuals with prior EPTB. CD39 expression is associated with active T regulatory cells and has been shown to be elevated during active TB disease (23). In the U.S. cohort we found no difference in median CD39 expression across the four study groups, but individuals with prior EPTB had the highest level of CD39 expression on Treg cells. In the larger Brazilian cohort, we found the same pattern, but demonstrated differences among groups with a high level of statistical significance. This suggests that a high frequency of circulating active Tregs is an immunological feature of individuals with successfully treated TB.

One hypothesis to explain increased susceptibility to EPTB would be some degree of immune deficiency. Individuals with HIV infection have a higher incidence of active TB disease, and a higher incidence of extrapulmonary disease (37), suggesting that a lack of pathogen-specific CD4+ T-cell responses may lead to more severe disease manifestations. Scriba et al. recently published data suggesting lower overall frequencies of Mtb-reactive T-cell responses in individuals with previously treated TB compared to individuals with LTBI, and these differing responses were dependent on the epitopes recognized (36). The cytokine profile of responding cells may be important for immune control. Arleham et al. found that individuals with a higher proportion of Mtb-reactive CD4+ T-cells with a TH1/TH17 cytokine profile were less likely to progress to active disease (38), and this population of cells appears to be preferentially depleted in the setting of HIV infection (39). However, the population of TH1/TH17 cells is still a small proportion of Mtb-reactive cells in these individuals, so it remains to be determined whether these cells are specifically responsible for immune control of LTBI.

The biggest limitation of our study was the small sample size. However, demonstration of similar results in two independent study populations decreased this concern. We measured systemic immune responses since they are likely to be a critical determinant of the extent and severity of disease. We measured immune responses specific to Mtb and did not assess responses to other microbial pathogens. However, immune responses to Mtb are likely most pertinent in TB pathogenesis. In addition, Th17 cells were not evaluated. Studies delineating immune cell sub-types could provide more insights into the pathogenesis of extrapulmonary TB. This could also provide insights into possible links between chronic inflammation and other immune-mediated diseases.

Despite our partial understanding of risk factors associated with progression from LTBI to active TB disease, it is unclear which immune responses are responsible for continued control of LTBI in the majority of infected individuals. Here we demonstrated that individuals with treated EPTB maintain higher frequencies of Mtb-reactive CD4+ memory T-cells compared with individuals with prior pulmonary disease or LTBI. These cells are “polyfunctional” with regard to their ability to generate IFN-γ and TNF-α in response to Mtb antigens. Consistent with our prior work, we found trends toward these individuals having higher frequencies of Tregs (13) as well higher Treg expression of CD39. It remains to be determined whether higher frequencies of Tregs or Treg CD39 expression was a feature of these individuals prior to active TB disease, and therefore increased their risk of developing EPTB. A goal of future longitudinal studies will be to identify individuals with LTBI prior to the development of active TB disease to determine whether there are immune signatures predictive of continued control of LTBI or that identify individuals at risk for disseminated disease.

## Data Availability Statement

The raw data supporting the conclusions of this article will be made available by the authors, without undue reservation.

## Ethics Statement

In the U.S. study, all participants provided written informed consent. The study was approved by the institutional review boards of Vanderbilt University Medical Center, Nashville Davidson Metro Public Health Department, and the Tennessee Department of Health. The study in Brazil was approved by the Maternidade Climério de Oliveira Ethics Committee, Federal University of Bahia. The patients/participants provided their written informed consent to participate in this study.

## Author Contributions

BB-D, TS, CF, AA, CN, RS, LB, CW, AB, JL, BA, and SK contributed to conception and design of the study. SK, BB-D, BA, and TS performed the data curation. BB-D, TS, CF, CN, LB, CW, and AB processed and analyzed the data. KS and BA worked on data visualization. BB-D, TS, SK, and BA wrote the first draft of the manuscript. All authors contributed to the article and approved the submitted version.

## Funding

The study was supported by grants from the National Institutes of Health, NIAID 1R21AI127129-01 (SK); NIH U01 AI069923 (CCASAnet and RePORT-Brazil: LB, AB, JL, BA, TS), NIAID 1 P30AI110527-03 Tennessee Center for AIDS Research (TNCFAR) (CN, CW, SK, TS); Brazilian Research Council/CNPq 469607/2014-9 (AA, JL), K23AI091692 (CF). The work from BA was supported by the Intramural Research Program of Fundação Oswaldo Cruz. BA and JL are senior scientists from CNPq. The work from LB was supported by the National Institutes of Health (U01 AI069923). BB-D received a research fellowship from the Coordenação de Aperfeiçoamento de Pessoal de Nível Superior (CAPES).

## Conflict of Interest

The authors declare that the research was conducted in the absence of any commercial or financial relationships that could be construed as a potential conflict of interest.

The handling editor declared a past co-authorship with the authors BA and TS.
