# Supplementary material for: Increased Frequency of Memory CD4+ T-Cell Responses in Individuals With Previously Treated Extrapulmonary Tuberculosis
Source: Front Immunol. 2020 Dec 17;11:605338. doi: 10.3389/fimmu.2020.605338 (PMC7774017; doi:10.3389/fimmu.2020.605338)
Supplement: Supplementary file 1 [file DataSheet_1.docx]

Increased frequency of memory CD4+ T-cell responses in individuals with previously treated extrapulmonary tuberculosis

Beatriz Barreto-Duarte, Timothy R. Sterling, Christina T. Fiske, Alexandre Almeida, Cynthia H. Nochowicz, Rita M. Smith, Louise Barnett, Christian Warren, Amondrea Blackman, Jose Roberto Lapa e Silva, Bruno B. Andrade, Spyros A. Kalams

Supplementary Material

**
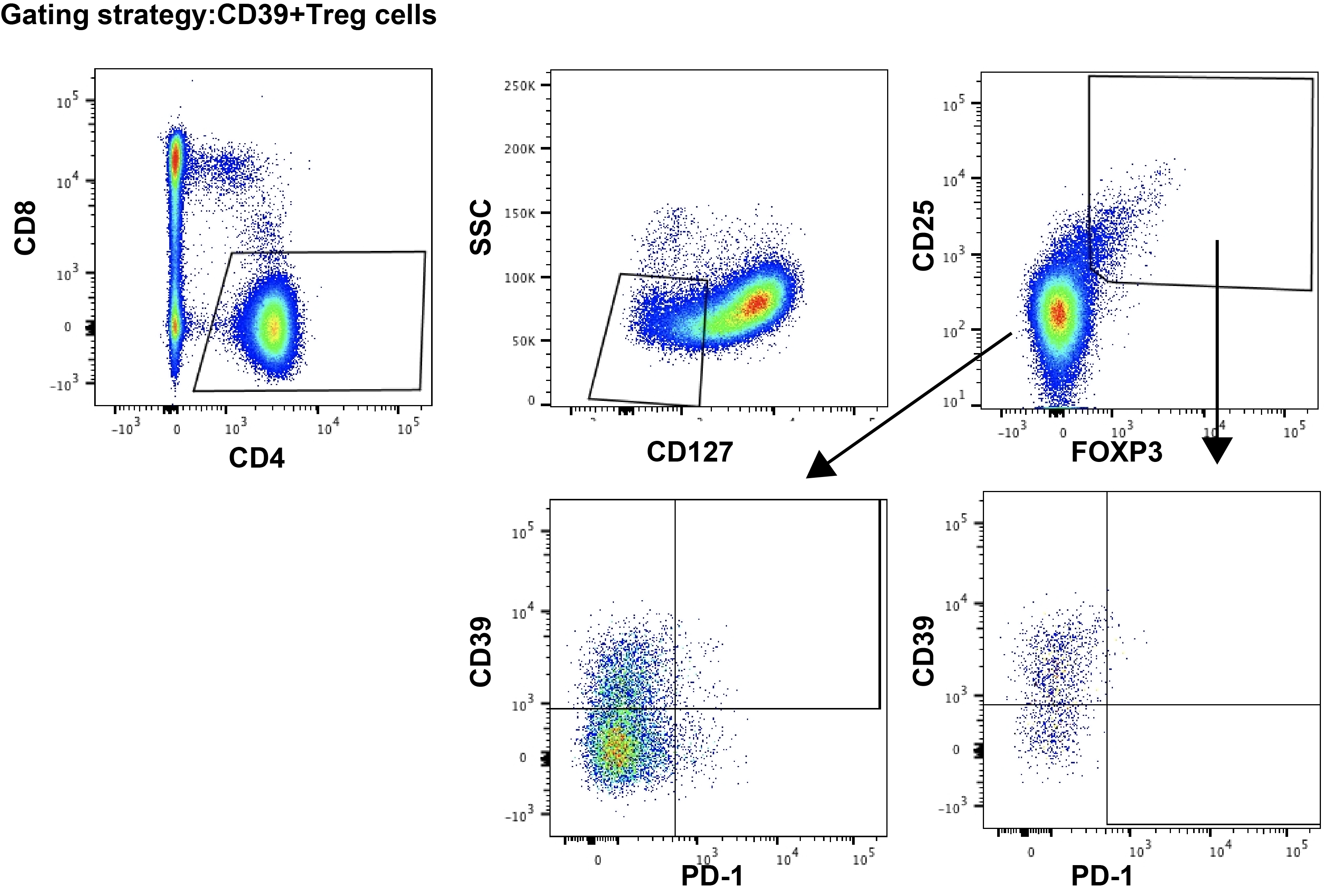
**

**Figure S1. Gating strategy for CD39+Treg cells.**


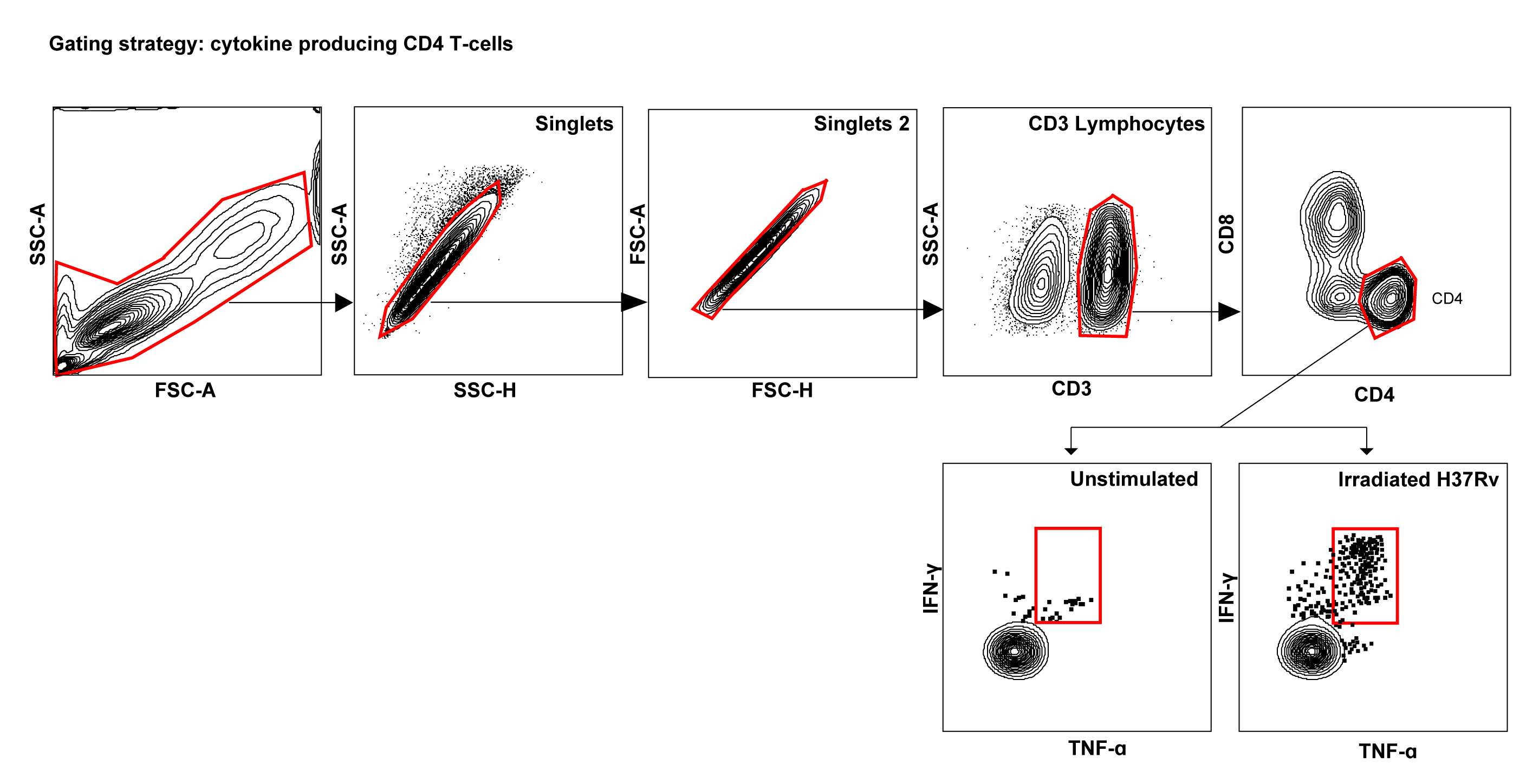


**Figure S2. Gating strategy for IFN-γ+TNF-α+CD4+ T cells.**
